# Elevated CO_2_ Impact on Common Wheat (*Triticum aestivum* L.) Yield, Wholemeal Quality, and Sanitary Risk

**DOI:** 10.1021/acs.jafc.0c02975

**Published:** 2020-08-31

**Authors:** Massimo Blandino, Franz-W. Badeck, Debora Giordano, Alessandra Marti, Fulvia Rizza, Valentina Scarpino, Patrizia Vaccino

**Affiliations:** † Department of Agricultural, Forest and Food Sciences (DISAFA), Università degli Studi di Torino, Largo P. Braccini 2, 10095 Grugliasco (TO), Italy; ‡ Consiglio per la ricerca in agricoltura e l’analisi dell’economia agraria, Research Centre for Genomics and Bioinformatics, via San Protaso 302, 29017 Fiorenzuola d’Arda, Italy; § Department of Food, Environmental and Nutritional Sciences (DeFENS), Università degli Studi di Milano, via G. Celoria 2, 20133 Milan, Italy; ∥ Consiglio per la ricerca in agricoltura e l’analisi dell’economia agraria, Research Centre for Cereal and Industrial Crops, S.S. 11 for Torino km 2,5, 13100 Vercelli, Italy

**Keywords:** Common wheat, carbon dioxide, FACE, grain yield, grain protein content, gluten aggregation, antioxidant compounds, mycotoxins

## Abstract

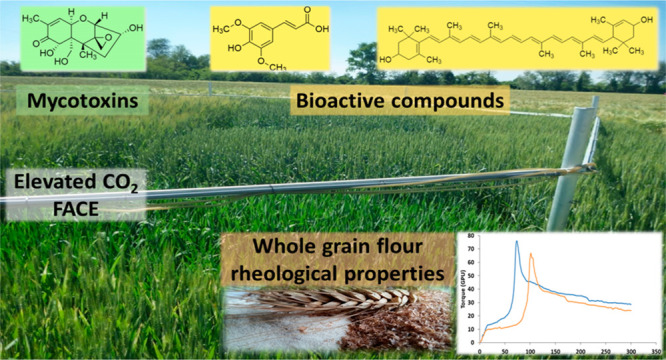

The rising atmospheric CO_2,_ concentration is expected to exert a strong impact on crop production, enhancing crop growth but threatening food security and safety. An improver wheat, a hybrid, and its parents were grown at elevated CO_2,_ e[CO_2_] in open field, and their yield and rheological, nutritional, and sanitary quality were assessed. For all cultivars, grain yield increased (+16%) and protein content decreased (−7%), accompanied by a reduction in dough strength. Grain nitrogen yield increased (+24%) only in ordinary bread making cultivars. e[CO_2_] did not result in significant changes in phenolic acid content and composition, whereas it produced a significant increase in the deoxynivalenol content. Different responses to e[CO_2_] between cultivars were found for yield parameters, while the effect on qualitative traits was quite similar. In the upcoming wheat cropping systems, agronomic practices and cultivar selection suited to guarantee higher nitrogen responsiveness and minimization of sanitary risk are required.

## Introduction

The release of carbon dioxide (CO_2_), methane (CH_4_), and nitrous oxide (N_2_O) due to human activities is one of the major causes of climatic changes with impacts on food security and safety for their effects on agricultural crops. The greenhouse gases are responsible for the increase in temperature, which will lead to higher drought stress for crops due to increased evapotranspiration combined with a more uneven distribution of rainfall events.^1^ Contrariwise, the expected increasing levels of atmospheric CO_2_ will increase photosynthesis and decrease transpiration and water use, leading to a productive advantage for crops that exhibit C3 photosynthetic metabolism, such as wheat and other small grain cereals.^2^ Several field experiments on wheat carried out in different growing areas through the application of free-air CO_2_ enrichment (FACE) led to a yield increase of 26% with an average CO_2_ level of 602 ppm^3^ due mainly to the increase in grain number per unit surface area rather than to the increase in kernel weight. Although many experiments have highlighted the wheat productive response to elevated CO_2_, the associated physiological mechanisms,^4,5^ and the interactions with crop practices^6−8^ and growing areas,^4,9^ few FACE studies have considered the effects on wheat cultivars (cv) with specific productive and qualitative traits. In this context, since tillering was reported to be the most important factor influencing yield at an elevated level of CO_2_,^5^ it would be interesting to investigate the response of cultivars with contrasting tillering capacity. Wheat hybrids, whose cultivation is expected to quickly increase in the near future, are planted at lower seeding rate compared to conventional varieties but can overcome the apparent initial disadvantage by means of a higher tillering capacity.^10^ Until now, only Yavad et al.^11^ have studied both a hybrid and a conventional wheat cultivar in a FACE experiment. However, this study was carried out in a subtropical climate, whereas no information is reported on the effect of FACE treatment on hybrid cultivars in temperate growing areas.

In addition to the yield effects, several FACE studies have highlighted the negative impact of elevated CO_2_ on wheat grain protein content (GPC) and baking quality.^6,12,13^ This negative effect could be even worse for high protein common wheat, which is classified, according to the specific classification terminology of different countries, as improver wheat (in Italy), excellent or class E wheat (in France and Germany), or Hard Red wheat (in the U.S.A.). Indeed, the optimum end-use quality and market price of these wheat categories are closely related to the protein content and to the dough rheological traits. In addition, FACE experiments showed that elevated CO_2_ can decrease the content of macro-, meso- and microelements and of essential amino acids in wheat grains, suggesting a decreased nutritional value of whole-meal wheat products in the future.^11,13,14^ However, no data are currently available on the effect of elevated CO_2_ on bioactive compounds such as phenolic acids (soluble and cell wall-bound forms) and xanthophylls (lutein and zeaxanthin). These compounds are responsible for the total antioxidant activity of wholemeal and result in numerous beneficial effects for the consumers, in particular for their protective anti-inflammatory effects.^15^

As opposed to these beneficial effects, the consumption of whole-grain products is associated with a potential high intake of contaminants, such as pesticides, heavy metals, and mycotoxins, all of which are more concentrated in the external kernel layers.^16^ Among mycotoxins, deoxynivalenol (DON) and its modified forms (DON-3-G, 3-ADON, 15-ADON) that belong to type-B trichothecenes, are frequently detected at harvest in wheat grain in temperate growing areas.^17^ These compounds are toxic for humans and animals and maximum admissible levels have been set up in several countries worldwide; therefore, it is useful to check the potential impact of elevated CO_2_ on the mycotoxin contamination of cereal grains under open field conditions.

Considering the interest in the topic, its complexity, and the knowledge gap stated above, the aim of the current work was to assess the impact of elevated CO_2_ concentration on common wheat yield and quality, considering a conventional high protein cultivar, a hybrid variety, and its parental lines. The impact on qualitative traits was focused specifically on the rheological, nutritional (e.g., content of antioxidant compounds), and sanitary (contamination by mycotoxins) wholemeal parameters. FACE experiments have been set up to investigate interactions between e[CO_2_] and growing seasons and between e[CO_2_] and the different genotypes. The objective was to provide further data on the response of common wheat in temperate areas under the future climatic scenarios in order to suggest some main objectives for wheat breeding.

## Materials and Methods

### Varieties Studied

The winter wheat (*Triticum aestivum spp. aestivum* L.) cv Bologna (S.I.S. Società Italiana Sementi, San Lazzaro di Savena, BO, Italy) was studied in three experimental years. According to the Italian bread wheat classification system,^18^ it is an improver wheat. In the third experimental year, the hybrid Hystar (Saaten-Union, Estrées St Denis, France, marketed in Italy by RV Venturoli, Pianoro, Italy) and its parents Apache (father, Limagrain, Saint Beauzire, France) and QH529 (mother, obtained from RV Venturoli, Pianoro, Italy) were also studied. All these cultivars are classified as ordinary bread-making wheat.

### Experimental Setup

Wheat plants were grown within the FACE facility of the Research Centre for Genomics and Bioinformatics (CREA-GB) at Fiorenzuola d’Arda (44.9278N, 9.8938E), Italy. The site is situated in the Po Valley at an elevation of 70 m.a.s.l. and has a warm continental climate, classified as Cfa (humid subtropical climate) in the Koeppen Geiger climate classification, that is, temperate climate without dry seasons and hot summers. The soil is alkaline (pH 8.09), with total carbonate, 10.19%; total C, 28.1 g kg^–1^; inorganic C, 12.22 g kg^–1^; organic C, 15.9 g kg^–1^; organic matter, 2.74%; total N, 0.10%; C/N ratio 15.6; P_2_O_5_, 21.7 mg kg^–1^; K_2_O 190 mg kg^–1^; and cation exchange capacity, 6.85 cmol(+) kg^–1^. Two different experiments have been carried out in order to analyze the effect of elevated carbon dioxide (e[CO_2_]) on wheat yield and qualitative traits compared to current ambient (a[CO_2_]). With the first experiment, cv Bologna was cultivated in three different years (Y1, 2011–12; Y2, 2012–13, Y3, 2015–16). The second experiment compares the four previously reported cultivars in Y3, according to a full factorial design. The experimental units for each cultivar were plots sized 2.2 m by 1.36 m. The FACE treatment with the e[CO_2_] target value set at 570 ppm was replicated in four octagons inscribed in circles of 14 m diameter. The a[CO_2_] controls were replicated four times in octagons without FACE at ambient CO_2_(404 ppm). For sowing, start of fumigation and harvest dates refer to Table 1. The FACE treatment was stopped when leaves were senescent and interrupted when the plots were covered with snow. The agronomic technique applied in the experimental trials was in accordance to the conventional farm management system in force in the experimental area. Briefly, the preceding crops are detailed in Table 1. The field was ploughed each year, incorporating the debris in the soil, and this was followed by disk harrowing to prepare a proper seedbed. Planting was conducted in 12 cm wide rows at a seeding rate of 350 seeds m^–2^, except for the hybrid cultivar and its parents, planted with a seeding rate of 200 seeds m^–2^. The field experiment received 30, 13, and 25 kg ha^–1^ of N, P, and K, respectively, at preseeding in Y1 and Y2. In Y3 preseeding fertilization was done with 45, 20, and 37 kg ha^–1^ of N, P and K, respectively. According to the ordinary management of the growing area, N was applied with two top-dressings at the tillering and stem elongation stages, as ammonium nitrate in Y1 and Y2, while in Y3 ammonium nitrate and ammonium sulfate were used for the first and second top-dressing, respectively. The total amount of nitrogen applied with the fertilizers was 149, 234, and 183 kg N ha^–1^ in Y1, Y2, and Y3, respectively. No fungicide was applied at flowering to control Fusarium head blight (FHB). Air temperature, precipitation, relative humidity, and global radiation were measured and recorded at 10 min intervals with an automatic meteorological station located within the field site of the FACE experiment at Fiorenzuola d’Arda.

**Table 1:** Meteorological and Agronomic Information of the Three-Year Experiment^a^

variable	Y1^b^	Y2	Y3
average daily mean temperature, sowing to heading [°C]	5.9 ± 0.11	6.1 ± 0.05	7.0 ± 0.02
average daily mean temperature, heading to harvest [°C]	20.6 ± 0.13	19.5 ± 0.04	19.6 ± 0.09
number of frost days	93	83	54
precipitation sum during the growth cycle [mm]	482	1027	347
precipitation sum sowing to heading [mm]^c^	399 ± 2.8	837 ± 1.2	320 ± 2.4
precipitation sum heading to harvest [mm]^c^	83 ± 2.8	191 ± 1.2	27 ± 2.4
potential evapotranspiration/precipitation	0.66	0.30	0.96
climatic water balance from heading to harvest [mm]	–93 ± 2.4	21 ± 1.5	–178 ± 1.6
cumulative water stress index from heading to harvest	0	0	27
preceding crop^d^	onion	wheat	wheat, oat, triticale
sowing date	Oct. 19, 2011	Oct. 24, 2012	Nov. 9, 2015
start of fumigation	Nov. 16, 2011	Nov. 9, 2012	Dec. 4, 2015
harvest date	Jul. 2, 2012	Jul. 11,2013	Jul. 12, 2016

a Climate traits that vary with heading date (mean ± standard deviation) are shown for cv. Bologna in experiment 1.

b Y1, 2011–12; Y2, 2012–13; Y3, 2015–16.

c Calculated using the heading dates of cv Bologna.

d In Y3, there were different preceding crops at the locations of the octagons.

### Morphological and Productive Traits

Average plant height per plot was measured during maturation. Ear density, that is, number of ears per square meter, was counted in the field (Y1) or determined on a 1.5 m linear meter harvest (Y2 and Y3). After harvesting by plot combine harvester (Nurserymaster, Wintersteiger, Austria) in Y1 or manually in Y2 and Y3, grains were threshed with the plot combine harvester and aboveground dry biomass, grain yield, and harvest index were determined. Biomass data are reported at dry mass basis.

### Grain Quality Characterization

Test weight (TW) was determined by means of a Dickey-John GAC2000 grain analysis meter (Dickey-John Corp. Auburn, IL, U.S.A.), according to the supplied program. Thousand kernel weight (TKW) was determined on two 100-kernel sets for each sample using an electronic balance.

Grain samples (500 g) from each plot were ground to wholemeal using a 1 mm sieve Cyclotec mill (Foss Tecator AB, Höganäs, Sweden). Protein content (PC) (N × 5.7, dry weight, AACC 39–10),^19^ and hardness (AACC 39–70)^19^ were determined by a NIR System Model 6500 (FOSS NIRSystems, Laurel, MD). Grain nitrogen yield (GNY), that is, nitrogen exported with the harvested grains, was calculated as grain yield × GPC/5.7 and expressed in kg N ha^–1^. The moisture content, determined in order to express all contents of bioactive compounds and mycotoxins on a dry weight (dw) basis, was obtained by oven-drying at 105 °C for 24 h.

### Technological Characterization

The SDS sedimentation volume (SSV) was determined according to Preston et al.^20^

The rheological properties were evaluated on wholemeal using GlutoPeak (Brabender GmbH and Co KG, Duisburg, Germany), according to the method reported by Marti et al.^21^ Briefly, flour (9 g) was dispersed in distilled water (10 mL), scaling both water and flour weight on a 14% flour moisture basis in order to keep the liquid-to-solid ratio constant. During the test, the sample and water temperature were maintained at 35 °C by circulating water through the jacketed sample cup. The paddle was set to rotate at 3000 rpm and each test was run for 500 s. Curves were elaborated using the software provided with the instrument (Brabender GlutoPeak v 2.1.2) and the following indices were considered: (i) maximum torque, expressed in Brabender equivalents (BE), which corresponds to the peak that occurs when gluten aggregates; (ii) peak maximum time (PMT), expressed in seconds, which corresponds to the peak torque time; and (iii) aggregation energy, expressed as the GlutoPeak Equivalent (GPE), which corresponds to the area under the portion of the curve 15 s before and 15 s after the peak. Each sample was analyzed in duplicate.

### Chemical Analyses

#### Chemicals

2,2-Diphenyl-1-picrylhydrazyl (DPPH), 2,6-di-*tert*-butyl-4-methylphenol (BHT, ≥ 99.0%), ethanol (CHROMASOLV, 99.8%), ethyl acetate (CHROMASOLV, 99.8%), hexane (CHROMASOLV, 97.0%), (±)-6-hydroxy-2,5,7,8-tetramethylchromane-2-carboxylic acid (Trolox, 97%), hydrochloric acid (HCl, 37.0%), methanol (CHROMASOLV, 99.9%), potassium hydroxide (KOH, 90.0%), sodium hydroxide (NaOH, ≥ 98.0%), *tert*-butyl methyl ether (MTBE, CHROMASOLV, 99.9%), *trans*-β-Apo-8′-carotenal, 2,4,6-tris(2-pyridyl)-s-triazine (TPTZ), and phenolic acid standards (caffeic acid ≥98%, *p*-coumaric acid ≥98%, *t*-ferulic acid ≥99%, *p*-hydroxybenzoic acid ≥99%, sinapic acid ≥98%, syringic acid ≥95%, and vanillic acid ≥97%) were purchased from Sigma-Aldrich (St. Louis, Missouri, U.S.). Xanthophylls standards (lutein ≥95% and zeaxanthin ≥98%) were purchased from Extrasynthese (Lyon, France).

Methanol (CH_3_OH), acetonitrile (CH_3_CN), and water (H_2_O) were LC gradient grade or LC-MS grade, depending on their use during the extraction or the analytical phases, and were purchased from VWR (Milan, Italy). Glacial acetic acid (CH_3_COOH) was obtained from Sigma-Aldrich (St. Louis, MO, U.S.A.). Mycotoxin standards were dissolved in acetonitrile (CH_3_CN) if not stated otherwise. Stock solutions of 3-acetyldeoxynivalenol (3-ADON), 15-acetyldeoxynivalenol (15-ADON), deoxynivalenol (DON), deoxynivalenol-3-glucoside (DON-3-G) in CH_3_CN/H_2_O 50/50, v/v, nivalenol (NIV) was purchased from Romer Laboratories Diagnostic GmbH (Tulln, Austria). Two composite standard working solutions were prepared by dissolving appropriate volumes of each analyte in a dilution phase mixture, CH_3_CN/H_2_O 50/50, v/v as follows: the first working solution contained DON and DON-3-G, whereas the second one contained 3-ADON, 15-ADON, and NIV. These two working solutions were then mixed in appropriate volumes and dissolved in CH_3_CN/H_2_O/CH_3_COOH 49.5/49.5/1 v/v/v in order to prepare the working solutions for the calibration. All of the solutions were stored at −20 °C in amber glass vials and were brought to room temperature before use.

### Extraction of the Soluble (SPAs) and Cell Wall-Bound Phenolic Acids (CWBPAs) and Quantification by Means of RP-HPLC/DAD

The extraction and quantification of soluble (free and conjugated) and cell wall-bound phenolic acids was performed according to the procedure proposed by Nicoletti et al.^22^ with some modifications as reported by Giordano et al.^23^ Quantifications were performed as reported in Giordano et al.^24^

### Extraction of Xanthophylls and Quantification by Means of RP-HPLC/DAD

The extraction and quantification of xanthophylls were performed as previously reported by Giordano et al.;^23^*trans*-β-Apo-8′-carotenal was used as internal standard to ensure that losses due to the extraction method were accounted for.

### Determination of Antioxidant Capacity through the DPPH Radical Scavenging Activity (AC_DPPH_)

DPPH radical scavenging activity of the flour (QUENCHER procedure–direct measurement on solid sample) was carried out as reported by Giordano et al.^24^ The DPPH radical scavenging activity was expressed as millimoles of Trolox equivalents/kg of sample (dw).

### Multimycotoxin LC-MS/MS Analysis

The extraction and sample preparation were performed by applying the dilute-and-shoot method reported by Scarpino et al.^25^ Briefly, 5 g of wheat flour was extracted by mechanical shaking at 300 rpm for 90 min with 20 mL of CH_3_CN/H_2_O/CH_3_COOH (79/20/1, v/v/v). The extract was filtered through Whatman grade 1 filters (Brentford, U.K.) and subjected to dilution with the same volume of diluting solution (CH_3_CN/H_2_O/CH3COOH 20/79/1, v/v/v). The diluted extract was vortexed and filtered through 15 mm diameter, 0.2 μm regenerated cellulose (RC) syringe filters (Phenex-RC, Phenomenex, Torrance, CA, U.S.A.) and 20 μL was analyzed without any further pretreatment. LC-MS/MS analysis was carried out on a Varian 310 triple quadrupole (TQ) mass spectrometer (Varian, Italy) equipped with an electrospray ionization (ESI) source, a 212 LC pump, a ProStar 410 AutoSampler and dedicated software. Liquid chromatography (LC) separation was performed on a Gemini-NX C_18_ 100 × 2.0 mm i.d., 3 μm particle size, 110 Å equipped with a C_18_ 4 × 2 mm security guard cartridge column (Phenomenex, Torrance, CA, U.S.A.). The mobile phase consisted of two eluents: water (eluent A) and methanol (eluent B), both of which were acidified with 0.1% v/v CH_3_COOH delivered at 200 μL min^–1^. The chromatographic conditions of the runs and the mass spectrometric parameters for the negative and positive ionization mode acquisitions were described in detail by Scarpino et al.^25^ The performance parameters of the method were reported for all the analyzed mycotoxins by Scarpino et al.^25^

### Statistical Analysis

Normal distribution and homogeneity of variances were verified by performing the Kolmogorov–Smirnov normality test and the Levene test, respectively. The analysis of variance (ANOVA) was conducted separately for each experiment in order to evaluate the effect of elevated carbon dioxide on grain yield, yield traits and qualitative traits on wholemeal using a completely randomized block design, in which the concentration of carbon dioxide and the year (experiment 1) and the concentration of carbon dioxide and the cultivar (experiment 2) were the independent variables. SPSS, Version 25.0 statistical package (SPSS Inc., Chicago), was used for the statistical analysis.

## Results

### Meteorological Conditions

The three growing seasons were characterized by contrasting thermal and hydrologic conditions (Table 1). The crops grown in Y1 experienced the coolest winter with the highest number of frost days and minimum temperatures down to −18.7 °C. However, during the nights with more severe frost the plants were protected by snow cover. With 54 frost days and minimum temperatures not decreasing below −7.4 °C, Y3 was characterized by the warmest winter. Thermal conditions in Y2 were intermediate with minimum temperatures reaching −11.2 °C, and plants were protected by snow cover during periods with more intense frost. In particular, three snow cover periods lasting for several days occurred during December, January, and February. Growing seasons in Y1, Y2, and Y3 were moderately wet, extremely wet, and relatively dry, respectively, with potential evapotranspiration not exceeding whole season precipitation in Y1 and Y2, while virtually identical to precipitation in Y3.

### Grain Yield and Yield Parameters

Aboveground biomass production and grain yield of cv Bologna increased in e[CO_2_] in the three years of experiment 1 (Table 2). CO_2_ X year interaction was significant for aboveground biomass: the percentage increase was 23.0%, 26.7% and 7.2% in Y1, Y2 and Y3, respectively. Conversely, the interaction was not significant for grain yield and ear density for which the average increase was 16.3% and 20.3%, respectively. The increase in grain yield and aboveground biomass is due to the higher plant density recorded in e[CO_2_]. Instead, TKW significantly decreased by 2.9% in e[CO_2_].

**Table 2:** Effect of FACE Treatment on Productive Parameters of Common Wheat

experiment	factor	source of variation	above ground biomass (t ha^–1^ d.m.)	plant height (cm)	grain yield (t ha^–1^ d.m.)	harvest index	ear density (n° m^–2^)	TKW^g^ (g)	GNY^g^ (kg N ha^–1^)
1^a^	CO_2_^b^	a[CO_2_]	14.0 b	82.0 b	6.7 b	0.49 a	693 b	37.5 a	167 a
		e[CO_2_]	16.7 a	84.1 a	7.8 a	0.47 a	874 a	36.4 b	181 a
		*P* (F)^c^	<0.001	<0.001	<0.001	0.073	<0.001	0.006	0.073
		sem^d^	0.2	2.6	0.1	0.024	218	1.7	32
	year^e^	Y1	19.3 a	86.9 a	8.7 a	0.45 b	784 a	33.9 c	193 a
		Y2	12.1 c	75.4 b	7.0 b	0.58 a	913 a	36.7 b	167 a
		Y3	14.6 b	86.9 a	6.0 c	0.41 c	558 b	40.4 a	166 a
		*P* (F)	<0.001	<0.001	<0.001	<0.001	<0.001	<0.001	0.079
		sem	0.2	3.2	0.1	0.03	267	2.1	39
	CO_2_ X year	*P* (F)	0.026	0.012	0.099	0.227	0.653	0.403	0.351
		sem	0.314	4.51	0.2	0.042	378	3.0	55
2^f^	CO_2_	a[CO_2_]	15.0 b	91.2 b	6.3 b	0.42 b	546 b	47.3 a	162 b
		e[CO_2_]	17.7 a	94.3 a	7.9 a	0.44 a	606 a	45.5 b	191 a
		*P* (F)	<0.001	<0.001	<0.001	0.013	0.017	0.008	<0.001
		sem	1.7	2.6	0.7	0.027	87	2.4	17
	cultivar (cv)	Bologna	14.6 b	86.9 c	6.0 b	0.41 b	579 a	40.4 d	166 b
		Apache	17.8 a	98.3 b	8.0 a	0.45 a	588 a	50.8 b	187 a
		QH529	14.3 b	82.0 d	5.8 b	0.41 b	579 a	44.7 c	138 c
		Hystar	18.7 a	103.9 a	8.4 a	0.45 a	559 a	55.6 a	189 a
		*P* (F)	<0.001	<0.001	<0.001	<0.001	0.842	<0.001	<0.001
		sem	2.4	3.6	0.9	0.038	107	3.4	24
	CO_2_X cv	*P* (F)	0.107	0.072	0.014	0.220	0.882	0.420	0.031
		sem	3.4	5.1	1.3	0.054	151	4.8	34

a Experiment carried out in three growing seasons on cv Bologna

b a[CO_2_] = ambient atmospheric carbon dioxide concentration, e[CO_2_] = elevated carbon dioxide concentration

c Means followed by different letters are significantly different (the level of significance is shown in the table). Reported values are based on four replications.

d sem: standard error of mean

e Y1, 2011–12; Y2, 2012–13; Y3, 2015–16

f Experiment carried out in the 2015–16 growing season

g TKW, thousand kernel weight; GNY, grain nitrogen yield

**Figure 1:**
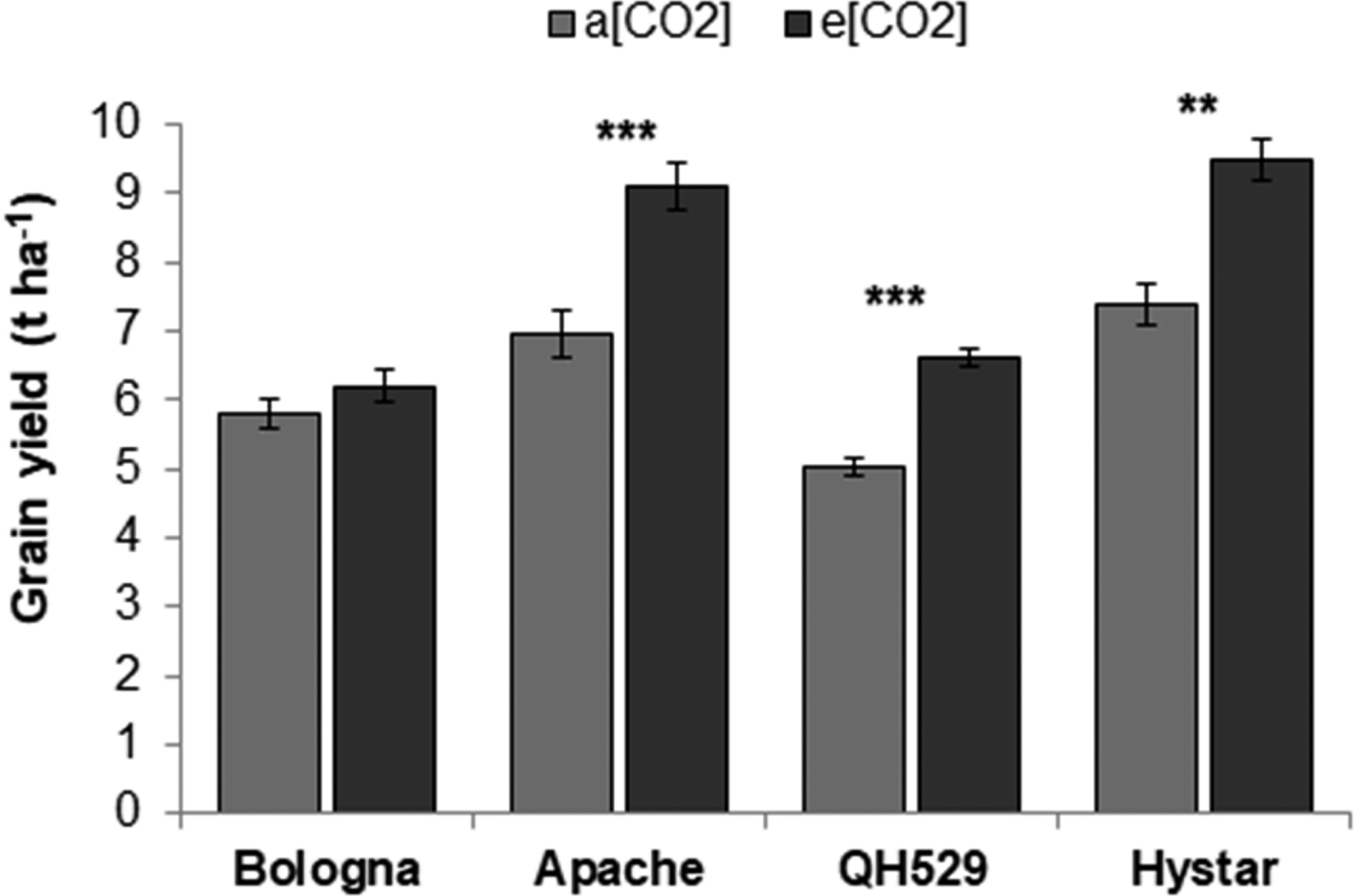
Effect of FACE treatment on grain yield of different common wheat cultivars. Experiment carried out in 2015-16 (Y3) on different cultivars (experiment 2). a[CO_2_] = ambient atmospheric carbon dioxide concentration, e[CO_2_] = elevated carbon dioxide concentration. Bars with asterisks are significantly different: *** P<;0.001; ** P< 0.01; * P<0.05. The error bars represent the standard error of means.

Plant height slightly increased under e[CO_2_] and was lower in Y2 than Y1 and Y3. The stimulation of height growth was most marked in Y2 (+6%). Harvest index did not change in response to e[CO_2_] with a slight, nonsignificant decreasing trend. The length of the vegetative growth period, that is, the number of days from sowing to heading, slightly increased but nonsignificantly by about 1 day in response to e[CO_2_]. It was 201 to 202 days in Y1 and Y2, while it was much shorter in Y3 (175 days). Aboveground biomass production, ear density, grain yield, harvest index, and plant height increased in e[CO_2_] relative to a[CO_2_] in experiment 2, while TKW significantly decreased (Table 2). However, the response to e[CO_2_] varied between cultivars. The grain yield increase was 31.1%, 28.5%, 31.2%, and 7.3% in Apache, Hystar, QH529, and Bologna, respectively, with a significant increase for Apache, QH529, and Hystar (Figure 1). The ear density increased by 12.7%, 10.3%, 6.3%, and 16.6% in Apache, Hystar, QH529, and Bologna, respectively. GNY did not differ significantly between years and treatments in experiment 1. In experiment 2, the treatment x cultivar interaction was significant with GNY in cv Bologna increasing only marginally by 0.4% in e[CO_2_], whereas GNY of Apache, Hystar, and QH529 increased significantly by 23.4, 22.8, and 25.3%, respectively.

### Grain and Wholegrain Flour Quality

TW and grain hardness were not affected by FACE treatment in both experiments (Table 3). Conversely, GPC significantly decreased under e[CO_2_] compared to a[CO_2_] (−7.0% and −6.1% on average in experiment 1 and 2, respectively). Although the experiments were carried out in years and with cultivars characterized by different GPC, no significant interaction between CO_2_ concentration and the considered factors was observed. SSV was not affected by FACE treatment in both experiments. Contrarily, CO_2_ concentration significantly affected the gluten aggregation properties of cv Bologna (experiment 1). In particular, e[CO_2_] promoted an increase in peak maximum time (PMT), indicating slower aggregation, and a decrease in both maximum torque (−12.5%) and aggregation energy (−10.7%), suggesting gluten weakening. As expected, also the year affected the gluten aggregation properties of wheat with Y3, characterized by the lowest grain yield, being significantly different from the others. Indeed, Y3 exhibited a lower aggregation time and the highest maximum torque and energy, suggesting the highest gluten strength, confirmed also by the highest SSV value (71 mL). No significant interaction between CO_2_ concentration and year was observed for gluten aggregation properties. The effect of e[CO_2_] on gluten aggregation kinetics was confirmed in the second experiment. QH529 and Hystar exhibited a similar GlutoPeak profile with an intermediate behavior between Bologna and Apache. The interaction between FACE treatment and cultivars was never significant, resulting in a similar impact on wholemeal rheological properties of wheat genotypes belonging to different qualitative market classes in both CO_2_ treatments.

**Table 3:** Effect of FACE Treatment on Grain Qualitative Traits and Rheological Parameters of Common Wheat Wholemeal

							glutopeak parameters		
experiment	factor	source of variation	TW^g^ (kg hl^–1^)	grain hardness	GPC^g^ (%)	SSV^g^ (mL)	PMT^g^ (s)	maximum torque (BE)^g^	aggregation energy (GPE)^g^
1^a^	CO_2_^b^	a[CO_2_]	81.9 a	70.3 a	14.2 a	61 a	90 b	64 a	1422 a
		e[CO_2_]	82.2 a	68.2 a	13.2 b	59 a	114 a	56 b	1270 b
		*P* (F)^c^	0.566	0.056	<0.001	0.314	<0.001	<0.001	<0.001
		sem^d^	2.2	4.9	0.6	6.8	19	5	87
	year^e^	Y1	82.7 a	70.0 a	12.7 c	54 b	108 a	56 b	1313 b
		Y2	83.0 a	69.7 a	13.5 b	53 b	115 a	54 b	1257 c
		Y3	80.6 b	68.3 a	15.8 a	71 a	86 b	68 a	1458 a
		*P* (F)	<0.001	0.406	<0.001	<0.001	<0.001	<0.001	<0.001
		sem	2.7	6.0	0.8	8.3	23	6	107
	CO_2_X year	*P*(F)	0.892	0.291	0.942	0.224	0.470	0.309	0.830
		sem	3.8	8.5	1.1	11.8	32	8	151
2^f^	CO_2_	a[CO_2_]	78.5 a	53.2 a	14.7 a	64 a	66 b	59 a	1281 a
		e[CO_2_]	78.8 a	52.8 a	13.8 b	61 a	80 a	53 b	1134 b
		*P* (F)	0.608	0.944	<0.001	0.074	<0.001	<0.001	0.003
		sem	2.4	4.2	0.7	4.7	11	4	203
	cultivar (cv)	Bologna	80.6 a	68.3 a	15.8 a	71 a	86 a	68 a	1458 a
		Apache	79.5 a	54.1 b	13.3 bc	65 b	58 b	54 b	1184 b
		QH529	73.0 b	33.1 d	13.5 b	48 c	68 b	43 c	1014 c
		Hystar	79.7 a	41.3 c	12.8 c	55 d	65 b	45 c	926 c
		*P* (F)	<0.001	<0.001	<0.001	<0.001	<0.001	<0.001	<0.001
		sem	3.4	5.9	1.0	6.6	16	6	288
	CO_2_ X cv	*P* (F)	0.712	0.400	0.438	0.117	0.387	0.224	0.409
		sem	4.8	8.3	1.4	9.4	22	9	407

a Experiment carried out in three growing seasons on cv Bologna.

b a[CO_2_] = ambient atmospheric carbon dioxide concentration, e[CO_2_] = elevated carbon dioxide concentration

c Means followed by different letters are significantly different (the level of significance is shown in the table). Reported values are based on four replications.

d sem: standard error of mean

e Y1, 2011–12; Y2, 2012–13; Y3, 2015–16

f Experiment carried out in the 2015–16 growing season

g TW, test weight; GPC, grain protein content; SSV, SDS sedimentation volume; PMT, peak maximum time; BE, Brabender equivalent; GPE, GlutoPeak equivalent.

### Bioactive Compound Content

**Figure 2:**
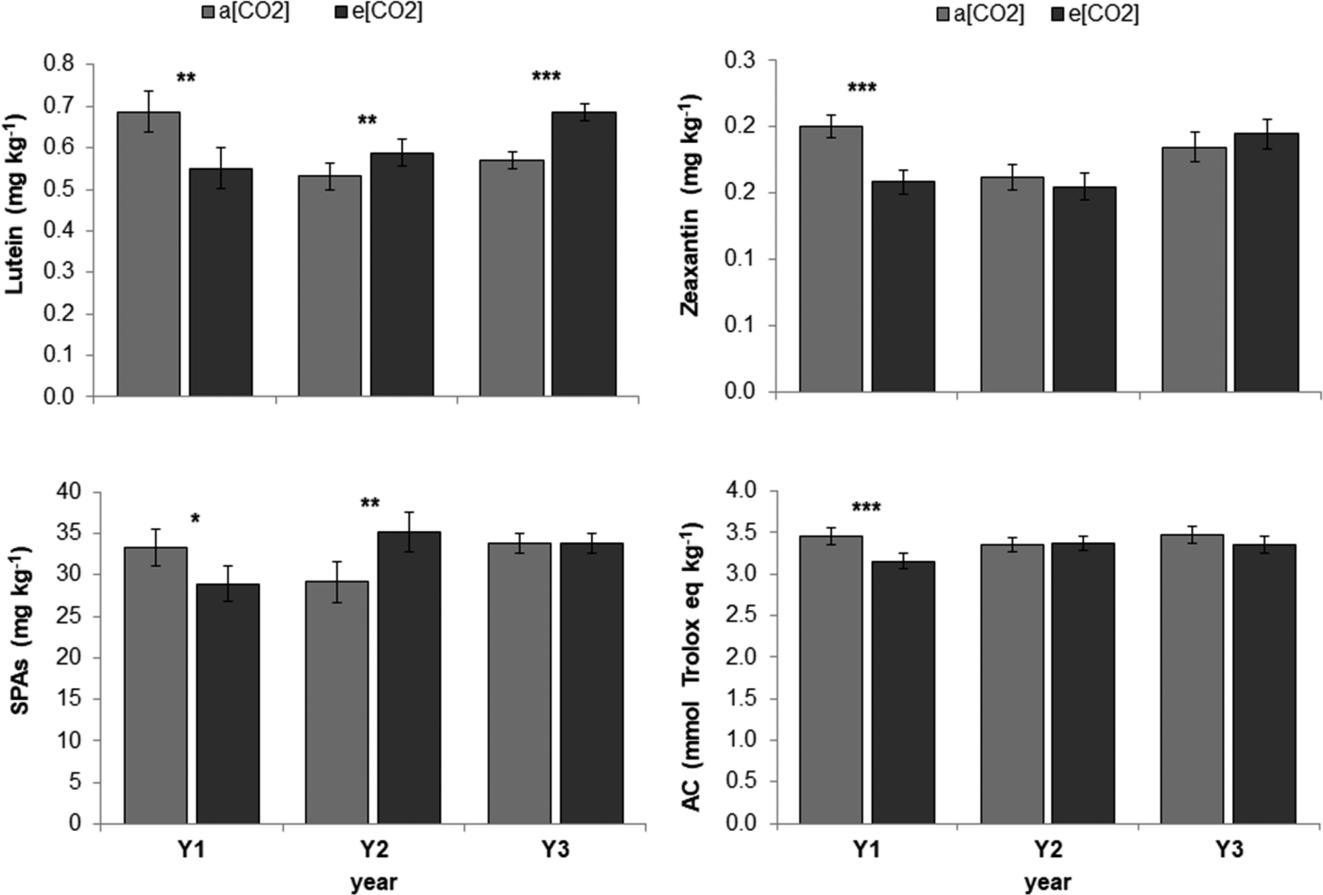
Effect of FACE treatment on the content of bioactive compounds and antioxidant capacity (AC) in common wheat wholemeal. Experiment carried out in 3 years (2011-12, 2012-13 and 2015-16) on cv Bologna (experiment 1). a[CO_2_] = ambient atmospheric carbon dioxide concentration, e[CO_2_] = elevated carbon dioxide concentration. Bars with asterisks are significantly different: *** P<0.001; ** P<0.01; * <;0.05. Data are expressed on a dw basis. The error bars represent the standard error of means.

In the first experiment, carried out on cv Bologna, the CO_2_ concentration did not significantly affect the content of bioactive compounds except for a slight but significant reduction of both zeaxanthin and antioxidant capacity in e[CO_2_] (Table 4). Whereas the total antioxidant capacity was constant among the years, the contents of several antioxidant compounds were different. A higher content of soluble sinapic acid and lower content of CWBPAs and bound ferulic acid were observed in Y3 compared to Y1 and Y2. Both lutein and zeaxanthin were the lowest in Y2. A significant interaction between FACE treatment and year was observed for SPAs, soluble sinapic acid, lutein, zeaxanthin, and for the AC_DPPH_ (Figure 2). Zeaxanthin and AC_DPPH_ showed significant differences between a[CO_2_] and e[CO_2_] only in Y1. Otherwise, lutein decreased in Y1, while it increased significantly both in Y2 and Y3. SPAs decreased in Y1 and increased in Y2, while no significant change was observed in Y3. In the second experiment, highly significant differences were observed among the cultivars for all compounds. Only lutein content was affected by e[CO_2_] with an increase of 9%. The interaction between FACE treatment and cultivars was never significant. Regardless the cultivar, the growing season and the FACE treatment, sinapic acid was the main soluble phenolic acid (58.1%; Figure 1S), followed by ferulic acid (21.0%) and vanillic acid (8.8.%). Ferulic acid (89.0%) was the predominant CWBPAs, followed by sinapic acid (5.9%), and *p*-coumaric acid (2.8%).

**Table 4:** Effect of FACE Treatment on the Content of Bioactive Compounds and Antioxidant Capacity (AC_DPPH_) in Common Wheat Wholemeal

			phenolic acids				xanthophylls		
experiment	factor	source of variation	SPAs^a^ (mg kg^–1^)	soluble sinapic acid (mg kg^–1^)	CWBPAs^a^ (mg kg^–1^)	bound ferulic acid (mg kg^–1^)	lutein (mg kg^–1^)	zeaxanthin (mg kg^–1^)	AC_DPPH_ (mmol Trolox eq kg^–1^)
1^b^	CO_2_^c^	a[CO_2_]	32.1 a	17.5 a	503.6 a	446.1 a	0.59 a	0.18 a	3.43 a
		e[CO_2_]	32.6 a	17.3 a	516.3 a	456.8 a	0.61 a	0.17 b	3.29 b
		*P* (F)^d^	0.594	0.736	0.434	0.459	0.441	0.005	0.002
		sem^e^	5.4	2.3	90.4	65.5	0.07	0.02	0.19
	year^f^	Y1	31.1 a	16.4 b	516.7 a	455.0 a	0.62 a	0.18 a	3.31 a
		Y2	32.2 a	16.8 b	539.6 a	479.8 a	0.56 b	0.16 b	3.36 a
		Y3	33.8 a	19.1 a	473.5 b	419.5 b	0.63 a	0.19 a	3.41 a
		*P* (F)	0.081	<0.001	0.006	0.005	0.003	0.000	0.123
		sem	7.7	2.9	127.8	80.2	0.09	0.02	0.23
	CO_2_X year	*P* (F)	<0.001	0.003	0.564	0.476	<0.001	<0.001	0.016
		sem	10.8	4.1	180.7	113.5	0.13	0.03	0.33
2^g^	FACE	a[CO_2_]	41.3 a	25.4 a	542.4 a	483.0 a	1.2 b	0.23 a	3.41 a
		e[CO_2_]	42.0 a	25.3 a	562.9 a	503.2 a	1.3 a	0.23 a	3.39 a
		*P* (F)	0.294	0.986	0.412	0.363	0.012	0.952	0.931
		sem	3.6	2.8	84.3	75.6	0.16	0.02	0.18
	cultivar (cv)	Bologna	33.8 d	19.1 c	473.5 b	419.5 b	0.63 d	0.19 b	3.41 ab
		Apache	37.8 c	21.2 c	611.0 a	548.6 a	2.20 a	0.26 a	3.27 b
		QH529	55.3 a	37.1 a	627.4 a	561.5 a	1.22 c	0.25 a	3.55 a
		Hystar	47.4 b	30.1 b	577.8 a	516.5 a	1.54 b	0.27 a	3.34 b
		*P* (F)	<0.001	<0.001	<0.001	<0.001	<0.001	<0.001	<0.001
		sem	5.1	3.9	119.2	107.0	0.23	0.04	0.26
	CO_2_ X cv	*P* (F)	0.823	0.956	0.510	0.521	0.630	0.449	0.173
		sem	7.2	5.6	168.6	151.3	0.32	0.05	0.37

a Sum of the soluble phenolic acids (SPAs) and cell wall-bound phenolic acids (CWBPAs) determined by means of the RP-HPLC/DAD.

b Experiment carried out in three growing seasons on cv Bologna.

c a[CO_2_] = ambient atmospheric carbon dioxide concentration, e[CO_2_] = elevated carbon dioxide concentration

d Means followed by different letters are significantly different (the level of significance *P* is shown in the table). Reported values are based on four replications. Data are expressed on a dw basis.

e sem: standard error of mean

f Y1, 2011–12; Y2, 2012–13; Y3, 2015–16

g Experiment carried out in the 2015–16 growing season

### Mycotoxin Content

**Figure 3:**
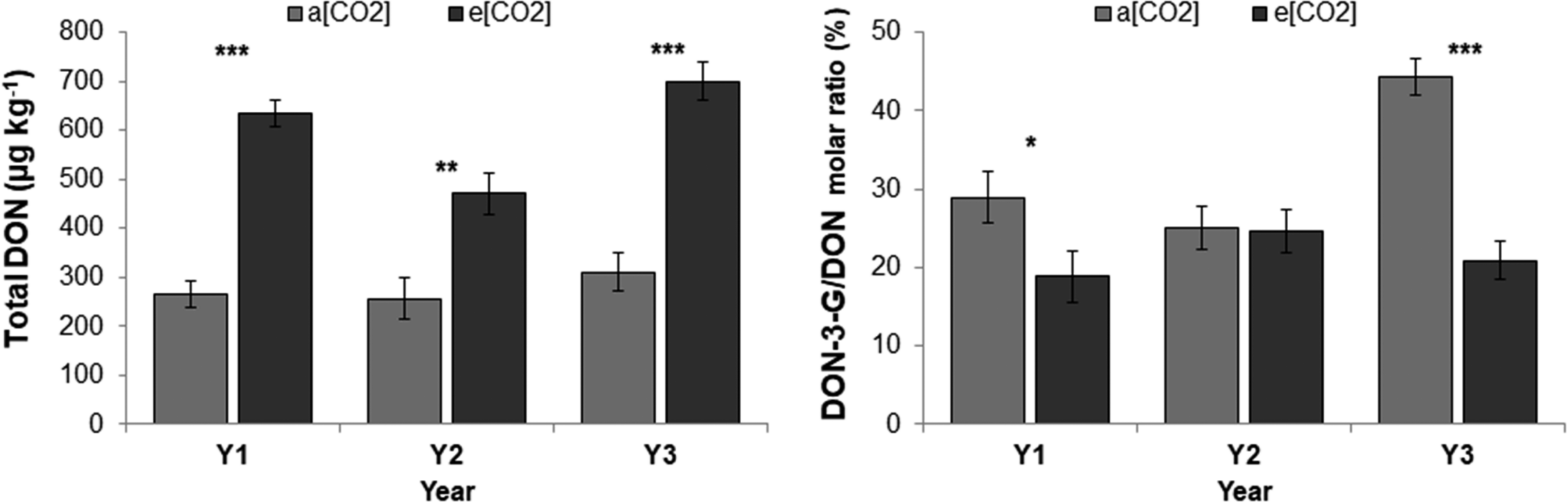
Effect of FACE treatment on total deoxynivalenol (DON) contamination in common wheat wholemeal. Experiment carried out in 3 growing seasons (2011-12, 2012-13 and 2015-16) on cv Bologna (1st experiment). a[CO_2_] = ambient atmospheric carbon dioxide concentration, e[CO_2_] = elevated carbon dioxide concentration. Bars with asterisks are significantly different: *** P<0.001; ** P<0.01; * P<0.05. The error bars represent the standard error of means.

The multimycotoxin LC–MS/MS analysis detected the trichothecenes DON and DON-3-G, while 3-ADON, 15-ADON, and NIV were under the limit of detection (LOD) for all samples. Y3 recorded the highest content of total DON (sum of DON, DON-3-G, 3-ADON, and 15-ADON), followed by Y1 and Y2 (Table 5). The DON-3-G/DON ratio was significantly higher in Y3 compared to Y1 and Y2. On average, e[CO_2_] resulted in a significant increase in total DON (+120%), DON (+146%), and DON-3-G (+64%). The DON-3-G/DON ratio was significantly reduced by 32% in e[CO_2_] compared to a[CO_2_]. The interaction between FACE treatment and year was significant (Figure 3): a higher increase in total DON content (*P* < 0.001, +133%) and a significant reduction in DON-3-G/DON ratio was observed in Y1 and Y3 under e[CO_2_] in comparison to a[CO_2_]. Conversely in Y2, the e[CO_2_] treatment significantly increased the total DON (+84%) but the DON-3-G/DON ratio was not affected.

**Table 5:** Effect of FACE Treatment on Mycotoxins Content in Common Wheat Wholemeal

factor	source of variation	total DON (μg kg^–1^)	DON (μg kg^–1^)	DON-3-G (μg kg^–1^)	DON-3-G/DON (μg kg^–1^)
CO_2_^a^	a[CO_2_]	274 b	185 b	89 b	32 a
	e[CO_2_]	602 a	456 a	146 a	22 b
	*P* (F)^b^	<0.001	<0.001	<0.001	<0.001
	sem^c^	85	80	25	7
year^d^	Y1	450 b	339 a	111 a	24 b
	Y2	364 c	264 b	99 b	25 b
	Y3	533 a	383 a	150 a	31 a
	*P* (F)	0.001	0.008	0.001	0.005
	sem	90	85	26	7
CO_2_ X year	*P* (F)	0.024	0.008	0.941	<0.001
	sem	146	139	43	11

a a[CO_2_] = ambient atmospheric carbon dioxide concentration, e[CO_2_] = elevated carbon dioxide concentration. Experiment carried out in three growing seasons on cv Bologna

b Means followed by different letters are significantly different (the level of significance *P* is shown in the table). Reported values are based on four replications. Data are expressed on a dw basis.

c sem: standard error of mean

d Y1, 2011–12; Y2, :2012–13; Y3, 2015–16. DON, deoxynivalenol; DON-3-G, deoxynivalenol-3-glucoside.

## Discussion

Wheat biomass and grain yield increased by 19% and 16%, respectively, as a consequence of the higher photosynthetic rate under e[CO_2_] conditions, consistent with the majority of earlier studies.^1,4^ In their meta-analysis of 95 FACE experiments, Broberg et al.^3^ stated an average increase of 22% for grain yield, supported by an increase of aboveground biomass (+25%), grain number (+23%), and grain mass (+2%), while harvest index remained unaffected. Our study on cv Bologna during three experimental years highlights a significant interaction CO_2_ X environmental conditions for aboveground biomass, whereas the increase in grain yield was consistent between growing seasons. Conversely, a marked interaction CO_2_ X cultivar was observed in Y3: cv Apache and the hybrid Hystar showed a higher grain yield than cv Bologna, related to both higher biomass production and higher harvest index. Furthermore, grain yield CO_2_ responsiveness varied substantially between the cultivars, ranging from +7.2% for cv Bologna to +28.5% for Hystar, and +31.1% for Apache. The results corroborate data of Fares et al.^26^ obtained on durum wheat in the same environments. Ziska^27^ reported a higher response to e[CO_2_] as a result of a greater tiller production and increase in ear density per unit surface area. Also for semiarid conditions, Maphosa et al.^28^ highlight that ear density may be the major determinant of cultivar response to CO_2_.

Compared with conventional cultivars, hybrids exhibit higher speed of tiller occurrence thus relatively higher growth rate. Despite the higher tillering capacity of Hystar, regarding the grain yield the experiment did not result in indications for a higher responsiveness to e[CO_2_] of the hybrid (+28.5%) compared to the most productive parent (+31.1%). Yadav et al.^11^ reported that a hybrid and a conventional cultivar responded similarly but to a different extent to CO_2_ treatments with the hybrid showing higher yield advantage compared to the conventional cv (+19% vs +11%) because of higher spike density. Liu et al.^29^ reported that hybrid rice appears to profit much more from e[CO_2_] than conventional rice, mainly for the significantly stronger effect on sink generation as indicated by a greater increase in spikelet number per unit surface area. In our experiment, the higher grain yield response to e[CO_2_] of both Hystar and Apache compared to Bologna and QH529 is a result of a greater tiller production.

Despite the importance of wheat as food and the elevated number of studies focusing on the effects of atmospheric CO_2_ on nitrogen and other macro-, meso-, and micronutrients, the knowledge of probable consequences of rising CO_2_ levels on its overall quality is still incomplete. Since quality requirements depend on wheat end-uses, the possible qualitative impact needs to be evaluated considering specific key parameters for the diverse supply chains (e.g., dough strength for improver wheat, phytochemicals for wholegrain flour, contaminants for baby foods). The present experiment resulted in the commonly observed drop of GPC under e[CO_2_],^13^ whereas grain TW and hardness did not change, maintaining unaltered the expected milling conditions and yield for common wheat. These data are in agreement with results reported by Panozzo et al.,^12^ whereas conflicting results for grain hardness were reported for previous FACE experiments.^6,7^ In our study, the average reduction of about 1 percentage point in GPC observed in the three year experiment for the improver wheat cv Bologna is consistent with the results obtained for ordinary bread-making cultivars in experiment 2, as well as with previous studies carried out in temperate growing areas.^6,13^ Panozzo et al.^12^ and Arachchige et al.^30^ reported a lower GPC reduction for ordinary bread-making wheat but confirm the absence of CO_2_ X genotype interaction in environments more prone to drought stress. Högy and Fangmeier^31^ hypothesized that at e[CO_2_] concentration GPC may decrease to values below the threshold for an adequate quality standard in bread-making (i.e., 11.5%). The present study highlights that the qualitative impact of near future air CO_2_ increase could be more marked for high protein common wheats, which are used in baking products that require high protein and dough strength.^18^ The achievement of GPC requirement (14%) for this marketing grade appears to be very challenging in a CO_2_ enriched atmosphere. Högy et al.^32^ reported a similar protein decrease (−1 percentage point) in an excellent baking quality spring cultivar.

Conversely to ear density, TKW decreased under e[CO_2_] providing indirect evidence that starch accumulation was not the cause of the decrease in GPC. This conclusion is consistent with the results reported by Tcherkez et al.^33^ for other wheat varieties grown in the Fiorenzuola FACE in Y3; in those samples, grain starch content decreased slightly (−4.9%) but significantly. Thus, it can be inferred that GPC mainly decreased because the increase in grain number exceeded the increase in GNY.

Because of the decrease in GPC, e[CO_2_] has a significant negative effect on bread-making performance resulting in lower sedimentation volume, higher mixing time, and lower bread volume.^13^ In the present study, the effect of CO_2_ concentration on wholemeal quality was assessed using a new high shear-based approach, that is, the GlutoPeak test, that has recently been proposed for the evaluation of gluten quality in refined^34^ and wholemeal^35^ flours. Usually, hard wheat flours (high protein) exhibit longer aggregation time (i.e., PMT) and higher maximum torque than flours of soft (low protein) wheat cultivars, as also found in our experiment 2 confronting cv Bologna with the hybrid and its parents (Table 2). The decrease in maximum torque under e[CO_2_], observed consistently in all the considered years and cultivars, coincides with the decrease in GPC. Fernando et al.^8^ reported that the effect of e[CO_2_] on mixograph peak height, a surrogate for dough strength, varied between grains grown under different environmental conditions but not between cultivars. Also in the present study, e[CO_2_] determined an increase in PMT and a decrease in aggregation energy in all environments, suggesting a consistent decrease in dough strength. Changes in gluten aggregation kinetics might be the result of the effects of e[CO_2_] on quality-related gluten protein fractions. Indeed, Wieser et al.^36^ observed a decrease in gliadins (by 20%), glutenins (by 15%), and glutenin macropolymer (by 19%) at increased atmospheric CO_2_ concentration. The high molecular weight (HMW) subunits of a high protein genotype were shown to be more affected than low molecular weight (LMW) ones.^32^ A higher decrease of HMW, compared to LMW glutenins, could contribute to a further decline in dough strength, especially for high protein cultivars; conversely, this variation could be beneficial for equilibrating the P/L ratio, often unbalanced toward excessive tenacity in high protein cvs.^37^ In addition to an adjustment of N rate according to the higher crop requirements, the future fertilization strategies could benefit from a shifting of N application timings from early (tillering) to late (from booting to anthesis) stages. Although a positive effect of elevated CO_2_ on postanthesis N uptake has been reported,^38^ a proper late season N fertilization could also contribute to guarantee an adequate GPC in a future climatic scenario,^37^ while selection for varieties with modified gluten protein fractions may contribute to maintaining baking quality.

At present, only few studies have investigated the effect of elevated atmospheric CO_2_ on the antioxidants of cereal grains and derived flour. The enrichment with [CO_2_] may differentially affect the content of phenolic compounds in cereal leaves. Li et al.^39^ observed an increase in total phenolics of wheat and maize leaves at e[CO_2_] during both the vegetative and the ripening stage. As far as antioxidants of rice grains are concerned, both free and bound phenolic compounds were negatively affected by e[CO_2_].^40^ The authors hypothesized that in response to CO_2_ enrichment the sink capacity of the grain is enhanced and carbon is diverted from being used in carbon-based secondary pathways. Although in the present study the FACE treatment did not result in a significant effect on SPAs and CWBPAs, the AC_DPPH_ of wholegrain flour decreased significantly following the exposure to e[CO_2_] in accordance with the results reported by Goufo et al.^40^ for brown rice. By comparing Bologna cv., the content of CWBPAs was lower in Y3, characterized by the highest TKW.

Xanthophylls are an important group of the carotenoids, whose members (e.g., lutein, zeaxanthin) have antioxidant effects, but the eCO_2_ effect on these pigments in wheat grain is still unknown.

A recent meta-analysis^41^ showed that carotenoids of vegetables were not affected by e[CO_2_]. On the contrary, in a second meta-analysis Loladze et al.^42^ observed that the overall effect of elevated CO_2_ on the content of plant carotenoids was significantly negative. The authors hypothesized that both active (downregulation of biosynthesis) and passive (dilution by carbohydrates) mechanisms could be responsible of the decrease in carotenoid content. Nevertheless, it is worth noting that the studies covered by their meta-analysis were mainly focused on carotenoids in leaves. In contrast, Zhang et al.^43^ observed an increase in total carotenoids of tomato fruits grown at e[CO_2_]. The increase was mainly ascribed to lycopene and β-carotene, whereas lutein showed less variation under e[CO_2_].

In the present study, lutein and zeaxanthin were affected differently by the FACE treatment. In the first experiment carried out on cv Bologna, a slight but significant reduction of zeaxanthin was observed under e[CO_2_]. Otherwise, the content of lutein was higher under e[CO_2_], even if the difference was not significant. In the second experiment, performed on 4 cv highly different for xanthophyll contents, plants grown under e[CO_2_] showed a significantly higher content of lutein, whereas the decrease of zeaxanthin was not significant. The differences observed on the functional impact of CO_2_, which varied in both plant species and plant organ considered, suggest that further studies will be necessary to confirm the effect of e[CO_2_] on cereal grain carotenoid content. In this context, it will be interesting to focus on cereals characterized by grain carotenoid content higher than that of common wheat, such as durum wheat, emmer, or the newly developed hybrid tritordeum.

Elevated CO_2_ caused more consistent effects on the content of mycotoxins, secondary metabolites produced by several fungal species, which have a broad spectrum of toxic actions. DON, a type-B trichothecene produced by *Fusarium* spp., is the most prevalent toxin in small grain cereals worldwide. To the best of the authors’ knowledge, this study is the first underlining an increasing risk of higher DON contamination in wheat due to e[CO_2_] in open field conditions with natural inoculum. All the cultivars compared in our study can be classified as moderately resistant to DON contamination. Previous investigations, such as those recently reported by Bencze et al.^44^ and Cuperlovic-Culf et al.,^45^ were carried out in controlled conditions (greenhouse or phytotron) and on *F. graminearum*- or *F. culmorum*-inoculated wheat. Cuperlovic-Culf et al.^45^ demonstrated that the effects of e[CO_2_] on FHB and DON contamination were dependent on both *F. graminearum* strain and wheat variety, underlining that moderately resistant lines may become significantly more susceptible to mycotoxin accumulation when infected by certain *F. graminearum* strains at e[CO_2_]. Similarly, Bencze et al.^44^ and Váry et al.^46^ observed variable effects of elevated CO_2_ on head blight between wheat varieties and suggested that CO_2_ has the potential to directly affect not only the fungal pathogen or the host plant but also the plant–pathogen interactions. Conversely, Vaughan et al.^47^ reported that e[CO_2_] increased maize susceptibility to *Fusarium verticillioides* proliferation, while fumonisin levels were unaltered. Maize simultaneously exposed to e[CO_2_] and drought was even more susceptible to *F. verticillioides* proliferation and also prone to higher levels of fumonisin contamination but the amount of fumonisin produced in relation to pathogen biomass remained lower than in corresponding plants grown at a[CO_2_].^48^ Therefore, the increase in fumonisin contamination in maize seemed to be likely due to greater pathogen biomass rather than to an increase in host-derived stimulants. As far as the aflatoxin risk due to the rising CO_2_ is concerned, Medina et al.^49^ have studied the response of *Aspergillus flavus* to climate change factors (water stress, temperature, and exposure to e[CO_2_]). Although growth was not significantly affected by the interaction between the involved environmental factors, the relative expression of genes in the biosynthetic pathway of aflatoxin production was stimulated by these interacting factors, resulting in an increase in phenotypic aflatoxin B_1_ production. Unfortunately, in the present study FHB symptoms in the ear were not recorded during grain filling. However, TW, which is strongly related to the severity of the disease, did not change in response to e[CO_2_]. This suggests that e[CO_2_] impacted more directly on the toxigenic capacity of fungal species responsible for mycotoxin contamination in grains, compared to the infection rate or the fungal development on wheat ears. Vaughan et al.^50^ suggested that rates of residue decomposition, *F. graminearum* inoculum production, and dispersal may be significantly altered by changes in atmospheric carbon dioxide concentration, temperature, and precipitation patterns, particularly in temperate climates. Thus, the results indicate that future environmental conditions, such as rising CO_2_ levels, may increase the threat of grain mycotoxins contamination. However, further studies are necessary to understand the overall impact of the CO_2_ increase on the development and the metabolism of fungal species responsible for FHB, considering also other emerging and still not yet regulated mycotoxins such as enniatins and moniliformin.^17^

In conclusion, our data underline that future wheat cultivation will require mitigation strategies in order to guarantee an adequate N soil uptake and control of head diseases. In order to counteract the negative effects of elevated CO_2_ on grain quality, the upcoming wheat cropping systems need to take into account all practices suited to maintain a higher soil fertility in parallel with the management of previous crop residues on the soil surface and the application of substances with high efficacy in controlling head fungal infection.^17^ Furthermore, since the simple use of more fertilizers and fungicides result in a further greenhouse gas emission, a more sustainable way to limit the impact of CO_2_ on wheat quality is the selection of adapted genotypes and their fundamental integration in cropping systems suitable to prevent the expected decline. Understanding the traits that can confer better adaptability to elevated CO_2_ is crucial for genetic improvement of both wheat productivity and quality. Breeding needs to focus on cultivars with higher tolerance to FHB in order to minimize the risk of mycotoxin contamination. The major negative impact of elevated CO_2_ could compromise particularly the cultivation and commercialization of high protein improver wheat. Particularly for this market category, it is necessary to develop cultivars with a higher N responsiveness, for example, characterized by greater soil uptake, due to a more extensive root system and/or superior sink capacity. The heterotic effects of wheat hybridization need to be explored for these potential qualitative benefits, in addition to the higher tiller and biomass production. Finally, research should focus on the interaction of genotypes with growing conditions and agricultural practices to correctly address the priority for breeding selection in order to maintain existing wheat quality standards and ensure global food security and safety.

## Supporting Information Available

Effect of FACE treatment on the relative occurrence of soluble (SPAs) and cell wall-bound (CWBPAs) phenolic acids in common wheat wholemeal (Figure S1) (PDF)

## ABBREVIATIONS

3-ADON: 3-acetyldeoxynivalenol
15-ADON: 15-acetyldeoxynivalenol
a[CO_2_]: atmospheric carbon dioxide concentration
ANOVA: analysis of variance
BE: Brabender equivalent
BHT: 2,6-di*tert*-butyl-4-methylphenol
CH_3_CN: acetonitrile
CH_3_COOH: glacial acetic acid
CH_3_OH: methanol
CH_4_: methane
CO_2_: carbon dioxide
CREA-GB: Research Centre for Genomics and Bioinformatics
CV: cultivar
CWBPAs: cell wall-bound phenolic acids
DON: deoxynivalenol
DON-3-G: deoxynivalenol-3-glucoside
DPPH: 2-diphenyl-1-picrylhydrazyl
DM: dry matter
DW: dry weight
e[CO_2_]: elevated carbon dioxide concentration
EC: European Commission
EFSA: European Food Safety Authority
ESI: electrospray ionization
FACE: free-air CO_2_ enrichment
FHB: Fusarium head blight
GNY: grain nitrogen yield
GPC: grain protein content
GPE: GlutoPeak equivalent
GS: growth stage
HCl: hydrochloric acid
H_2_O: water
KOH: potassium hydroxide
LC-MS/MS: liquid chromatography coupled with tandem mass spectrometry detection
LOD: limit of detection
MTBE: *tert*-butyl methyl ether
NaOH: sodium hydroxide
N_2_O: nitrous oxide
NIV: nivalenol
PMT: peak maximum time
REGW-Q test: Ryan/Einot and Gabriel/Welsch test
SDS: sodium dodecyl sulfate
S.I.S.: Società Italiana Sementi
SPAs: soluble phenolic acids
SSV: SDS sedimentation volume
TE: Trolox equivalents
TKW: thousand kernel weight
TPTZ: 2,4,6-tris(2-pyridyl)-*s*-triazine
Trolox: (±)-6-hydroxy-2,5,7,8-tetramethylchromane-2-carboxylic acid
TW: test weight

## References

[ref1] Korres N. E.; Norsworthy J. K.; Tehranchian P.; Gitsopoulos T. K.; Loka D. A.; Oosterhuis D. M.; Gealy D. R.; Moss S. R.; Burgos N. R.; Miller M. R.; Palhano M. Cultivars to face climate change effects on crops and weeds: a review. Agron. Sustainable Dev. 2016, 36, 12 10.1007/s13593-016-0350-5.

[ref2] Ainsworth E. A.; Rogers A. The response of photosynthesis and stomatal conductance to rising [CO_2_]: mechanisms and environmental interactions. Plant, Cell Environ. 2007, 30, 258–270. 10.1111/j.1365-3040.2007.01641.x. 17263773

[ref3] Broberg M. C.; Högy P.; Feng Z.; Pleijel H. Effects of elevated CO_2_ on wheat yield: non-linear response and relation to site productivity. Agronomy 2019, 9, 243 10.3390/agronomy9050243.

[ref4] Wang L.; Feng Z.; Schjoerring J. K. Effects of elevated atmospheric CO_2_ on physiology and yield of wheat (*Triticum aestivum* L.): a meta-analytic test of current hypotheses. Agric., Ecosyst. Environ. 2013, 178, 57–63. 10.1016/j.agee.2013.06.013.

[ref5] Dubey S. K.; Tripathi S. K.; Pranuthi G. Effect of elevated CO_2_ on wheat crop: mechanism and impact. Crit. Rev. Environ. Sci. Technol. 2015, 45 (21), 2283–2304. 10.1080/10643389.2014.1000749.

[ref6] Erbs M.; Manderscheid R.; Jansen G.; Seddig S.; Pacholski A.; Weigel H.-J. Effect of free-air CO2 enrichment and nitrogen supply on grain quality parameters and elemental composition of wheat and barley grown in a crop rotation. Agric., Ecosyst. Environ. 2010, 136, 59–68. 10.1016/j.agee.2009.11.009.

[ref7] Fernando N.; Panozzo J.; Tausz M.; Norton R. M.; Neumann N.; Fitzgerald G. J.; Seneweera S. Elevated CO2 alters grain quality of two bread wheat cultivars grown under different environmental conditions. Agric., Ecosyst. Environ. 2014, 185, 24–33. 10.1016/j.agee.2013.11.023.

[ref8] Fernando N.; Panozzo J.; Tausz M.; Norton R. M.; Fitzgerald G. J.; Myers S.; Nicolas M. E.; Seneweera S. Intra-specific variation of wheat grain quality in response to elevated [CO2] at two sowing times under rain-fed and irrigation treatments. J. Cereal Sci. 2014, 59, 137–144. 10.1016/j.jcs.2013.12.002.

[ref9] Jin J.; Armstrong R.; Tang C. Impact of elevated CO_2_ on grain nutrient concentration varies with crops and soils - a long term FACE study. Sci. Total Environ. 2019, 651 (2), 2641–2647. 10.1016/j.scitotenv.2018.10.170. 30463119

[ref10] Milan M.; Fogliatto S.; Blandino M.; Vidotto F. Are wheat hybrids more affected by weed competition than conventional cultivars?. Agronomy 2020, 10, 526 10.3390/agronomy10040526.

[ref11] Yadav A.; Bhatia A.; Yadav S.; Kumar V.; Singh B. The effect of elevated CO_2_ and elevated O3 exposure on plant growth, yield and quality of grain of two wheat cultivars grown in north India. Heliyon 2019, 5, e02317 10.1016/j.heliyon.2019.e02317. 31463405 PMC6710491

[ref12] Panozzo J. F.; Walker C. K.; Partington D. L.; Neumann N. C.; Tausz M.; Seneweera S.; Fitzgerald G. J. Elevated carbon dioxide changes grain protein concentration and composition and compromises baking quality. A FACE study. J. Cereal Sci. 2014, 60, 461–470. 10.1016/j.jcs.2014.08.011.

[ref13] Broberg M. C.; Högy P.; Pleijel H. CO_2_-induced changes in wheat grain composition: meta-analysis and response functions. Agronomy 2017, 7, 32 10.3390/agronomy7020032.

[ref14] Beleggia R.; Fragasso M.; Miglietta F.; Cattivelli L.; Menga V.; Nigro F.; Pecchioni N.; Fares C. Mineral composition of durum wheat grain and pasta under increasing atmospheric CO2 concentrations. Food Chem. 2018, 242, 53–61. 10.1016/j.foodchem.2017.09.012. 29037725

[ref15] Adom K. K.; Liu R. H. Antioxidant activity of grains. J. Agric. Food Chem. 2002, 50, 6182–6187. 10.1021/jf0205099. 12358499

[ref16] Sovrani V.; Blandino M.; Scarpino V.; Reyneri A.; Coisson J. D.; Travaglia F.; Locatelli M.; Bordiga M.; Montella R.; Arlorio M. Bioactive compound content, antioxidant activity, deoxynivalenol and heavy metal contamination of pearled wheat fractions. Food Chem. 2012, 135, 39–46. 10.1016/j.foodchem.2012.04.045.

[ref17] Blandino M.; Scarpino V.; Sulyok M.; Krska R.; Reyneri A. Effect of agronomic programmes with different susceptibility to deoxynivalenol risk on emerging contamination in winter wheat. Eur. J. Agron. 2017, 85, 12–24. 10.1016/j.eja.2017.01.001.

[ref18] Foca G.; Ulrici A.; Corbellini M.; Pagani M. A.; Lucisano M.; Franchini G. C.; Tassi L. Reproducibility of the Italian ISQ method for quality classification of bread wheats: An evaluation by expert assessors. J. Sci. Food Agric. 2007, 87, 839–846. 10.1002/jsfa.2785.

[ref19] AACC. Approved Methods of the American Association of Cereal Chemists, 10th ed.; The Association: St Paul, MN, 2000.

[ref20] Preston K. R.; March P. R.; Tipples K. H. An assessment of SDS-sedimentation test for prediction of Canadian bread wheat quality. Can. J. Plant Sci. 1982, 62, 545–553. 10.4141/cjps82-083.

[ref21] Marti A.; Cecchini C.; D’Egidio M. G.; Dreisoerner J.; Pagani M. A. Characterization of durum wheat semolina by means of a rapid shear-based method. Cereal Chem. 2014, 91, 542–547. 10.1094/CCHEM-10-13-0224-R.

[ref22] Nicoletti I.; Martini D.; De Rossi A.; Taddei F.; D’Egidio M. G.; Corradini D. Identification and quantification of soluble free, soluble conjugated, and insoluble bound phenolic acids in durum wheat (*Triticum turgidum* L. var. *durum*) and derived products by RP-HPLC on a semimicro separation scale. J. Agric. Food Chem. 2013, 61, 11800–11807. 10.1021/jf403568c. 24175612

[ref23] Giordano D.; Reyneri A.; Locatelli M.; Coisson J. D.; Blandino M. Distribution of bioactive compounds in pearled fractions of tritordeum. Food Chem. 2019, 301, 125228 10.1016/j.foodchem.2019.125228. 31377613

[ref24] Giordano D.; Beta T.; Vanara F.; Blandino M. Influence of agricultural management on phytochemicals of colored corn genotypes (*Zea mays* L.) - Part I: Nitrogen fertilization. J. Agric. Food Chem. 2018, 66 (17), 4300–4308. 10.1021/acs.jafc.8b00325. 29641199

[ref25] Scarpino V.; Reyneri A.; Blandino M. Development and Comparison of Two Multiresidue Methods for the Determination of 17 *Aspergillus* and *Fusarium* Mycotoxins in Cereals Using HPLC-ESI-TQ-MS/MS. Front. Microbiol. 2019, 10 (361), 1–12. 10.3389/fmicb.2019.00361. 30886605 PMC6409351

[ref26] Fares C.; Menga V.; Badeck F.; Rizza F.; Miglietta F.; Zaldei A.; Codianni P.; Iannucci A.; Cattivelli L. Increasing atmospheric CO_2_ modifies durum wheat grain quality and pasta cooking quality. J. Cereal Sci. 2016, 69, 245–251. 10.1016/j.jcs.2016.03.016.

[ref27] Ziska L. H. Three-year field evaluation of early and late 20th century spring what cultivars to projected increases in atmospheric carbon dioxide. Field Crop Res. 2008, 108, 54–59. 10.1016/j.fcr.2008.03.006.

[ref28] Maphosa L.; Fitzgerald G. J.; Panozzo J.; Partington D.; Walker C.; Kant S. Genotypic response of wheat under semi-arid conditions showed no specific responsive traits when grown under elevated CO_2_. Plant Prod. Sci. 2019, 22 (3), 333–344. 10.1080/1343943X.2019.1626254.

[ref29] Liu H.; Yang L.; Wang Y.; Huang J.; Zhu J.; Yunxia W.; Dong G.; Liu G. Yield formation of CO_2_-enriched hybrid rice cultivar Shanyou 63 under fully open-air field conditions. Field Crops Res. 2008, 108, 93–100. 10.1016/j.fcr.2008.03.007.

[ref30] Arachchige P. M. S.; Ang C.-S.; Nicolas M. E.; Panozzo J.; Fitzgerald G.; Hirotsu N.; Seneweera S. Wheat (*Triticum aestivum* L.) grain proteome response to elevated (CO_2_) varies between genotypes. J. Cereal Sci. 2017, 75, 151–157.

[ref31] Högy P.; Fangmeier A. Effect of elevated atmospheric CO_2_ on grain quality of wheat. J. Cereal Sci. 2008, 48, 580–591. 10.1016/j.jcs.2008.01.006.

[ref32] Högy P.; Brunnbauer M.; Koehler P.; Schwadorf K.; Breuer J.; Franzaring J.; Zhunusbayeva D.; Fangmeier A. Grain quality characteristics of spring wheat (*Triticum aestivum*) as affected by free-air CO_2_ enrichment. Environ. Exp. Bot. 2013, 88, 11–18. 10.1016/j.envexpbot.2011.12.007.

[ref33] Tcherkez G.; Ben Mariem S.; Larraya L.; García-Mina J. M.; Zamarreño A. M.; Paradela A.; Cui J.; Badeck F.-W.; Meza D.; Rizza F.; Bunce J.; Han X.; Tausz-Posch S.; Cattivelli L.; Fangmeier A.; Aranjuelo I. Despite minimal effects on yield, elevated CO_2_ has concurrent effects on leaf and grain metabolism in wheat. J. Exp. Bot. 2020, 10.1093/jxb/eraa330. PMC7751139 32687190

[ref34] Marti A.; Ulrici A.; Foca G.; Quaglia L.; Pagani M. A. Characterization of common wheat flours (*Triticum aestivum* L.) through multivariate analysis of conventional rheological parameters and gluten peak test indices. LWT-Food Sci. Technol. 2015, 64, 95–103. 10.1016/j.lwt.2015.05.029.

[ref35] Malegori C.; Grassi S.; Ohm J. B.; Anderson J.; Marti A. Gluto Peak profile analysis for wheat classification: skipping the refinement process. J. Cereal Sci. 2018, 79, 73–79. 10.1016/j.jcs.2017.09.005.

[ref36] Wieser H.; Manderscheid R.; Erbs M.; Weigel H. J. Effects of elevated atmospheric CO_2_ concentrations on the quantitative protein composition of wheat grain. J. Agric. Food Chem. 2008, 56 (15), 6531–6535. 10.1021/jf8008603. 18598044

[ref37] Blandino M.; Visioli G.; Marando S.; Marti A.; Reyneri A. Impact of late-season N fertilisation strategies on the gluten content and composition of high protein wheat grown under humid Mediterranean conditions. J. Cereal Sci. 2020, 94, 102995 10.1016/j.jcs.2020.102995.

[ref38] Dier M.; Siskora J.; Erbs M.; Weigel H. J.; Zörb C.; Manderscheid R. Positive effects of free air CO_2_ enrichment on N remobilization and post-anthesis uptake in winter wheat. Field Crops Res. 2019, 234, 107–118. 10.1016/j.fcr.2019.02.013.

[ref39] Li G.; Shi Y.; Chen X. Effect of elevated CO_2_ and O_3_ on phenolic compounds in spring wheat and maize leaves. Bull. Environ. Contam. Toxicol. 2008, 81, 436–439. 10.1007/s00128-008-9516-4. 18781273

[ref40] Goufo P.; Pereira J.; Figueiredo N.; Oliveira M. B. P. P.; Carranca C.; Rosa E. A. S.; Trindade H. Effect of elevated carbon dioxide (CO_2_) on phenolic acids, flavonoids, tocopherols, tocotrienols, γ-oryzanol and antioxidant capacities of rice (*Oryza sativa* L.). J. Cereal Sci. 2014, 59 (1), 15–24. 10.1016/j.jcs.2013.10.013.

[ref41] Dong J.; Gruda N.; Lam S. K.; Li X.; Duan Z. Effects of Elevated CO_2_ on Nutritional Quality of Vegetables: A Review. Front. Plant Sci. 2018, 9 (924), 1–11. 10.3389/fpls.2018.00924. 30158939 PMC6104417

[ref42] Loladze I.; Nolan J. M.; Ziska L. H.; Knobbe A. R. Rising atmospheric CO_2_ lowers concentrations of plant carotenoids essential to human health: a meta-analysis. Mol. Nutr. Food Res. 2019, 63, 1801047 10.1002/mnfr.201801047. 31250968

[ref43] Zhang Z. M.; Liu L. H.; Zhang M.; Zhang Y. S.; Wang Q. M. Effect of carbon dioxide enrichment on health-promoting compounds and organoleptic properties of tomato fruits grown in greenhouse. Food Chem. 2014, 153, 157–163. 10.1016/j.foodchem.2013.12.052. 24491715

[ref44] Bencze S.; Puskás K.; Vida G.; Karsai I.; Balla K.; Komáromi J.; Veisz O. Rising atmospheric CO_2_ concentration may imply higher risk of *Fusarium* mycotoxin contamination of wheat grains. Mycotoxin Res. 2017, 33, 229–236. 10.1007/s12550-017-0281-2. 28573418

[ref45] Cuperlovic-Culf M.; Vaughan M. M.; Vermillion K.; Surendra A.; Teresi J.; McCormick S. P. Effects of Atmospheric CO_2_ Level on the Metabolic Response of Resistant and Susceptible Wheat to *Fusarium graminearum* Infection. Mol. Plant-Microbe Interact. 2019, 32 (4), 379–391. 10.1094/MPMI-06-18-0161-R. 30256178

[ref46] Váry Z.; Mullins E.; McElwain J. C.; Doohan F. M. The severity of wheat diseases increases when plants and pathogens are acclimatized to elevated carbon dioxide. Glob. Change Biol. 2015, 21, 2661–2669. 10.1111/gcb.12899. 25899718

[ref47] Vaughan M. M.; Huffaker A.; Schmelz E. A.; Dafoe N. J.; Christensen S.; Sims J.; Martins V. F.; Swerbilow J.; Romero M.; Alborn H. T.; Allen L. H.; Teal P. E. A. Effects of elevated [CO2] on maize defence against mycotoxigenic *Fusarium verticillioides*. Plant, Cell Environ. 2014, 37, 2691–2706. 10.1111/pce.12337. 24689748 PMC4278449

[ref48] Vaughan M. M.; Huffaker A.; Schmelz E. A.; Dafoe N. J.; Christensen S. A.; McAuslane H. J.; Alborn H. T.; Allen L. H.; Teal P. E. A. Interactive Effects of Elevated [CO_2_] and Drought on the Maize Phytochemical Defense Response against Mycotoxigenic *Fusarium verticillioides*. PLoS One 2016, 11 (7), e0159270 10.1371/journal.pone.0159270. 27410032 PMC4943682

[ref49] Medina A.; Rodrıguez A.; Sultan Y.; Magan N. Climate change factors and *A. flavus*: effects on gene expression, growth and aflatoxin production. World Mycotoxin J. 2015, 8, 171–179. 10.3920/WMJ2014.1726.

[ref50] Vaughan M. M.; Backhouse D.; Del Ponte E. M. Climate change impacts on the ecology of *Fusarium graminearum* species complex and susceptibility of wheat to Fusarium head blight: A review. World Mycotoxin J. 2016, 9, 685–700. 10.3920/WMJ2016.2053.

