# Supplementary material for: Elevated CO2 Impact on Common Wheat (Triticum aestivum L.) Yield, Wholemeal Quality, and Sanitary Risk
Source: J Agric Food Chem. 2020 Aug 31;68(39):10574–85. doi: 10.1021/acs.jafc.0c02975 (PMC8011921; doi:10.1021/acs.jafc.0c02975)
Supplement: Supplementary file 1 — jf0c02975_si_001.pdf [file jf0c02975_si_001.pdf]

SUPPORTING INFORMATION FOR PUBLICATION

Elevated CO2 impact on common wheat (*Triticum aestivum* L.) yield, wholemeal quality and sanitary risk

AUTHORS

Massimo Blandino[a*], Franz-W. Badeck[b], Debora Giordano[a], Alessandra Marti[c], Fulvia Rizza[b], Valentina Scarpino[a], Patrizia Vaccino[d]

AFFILIATIONS

[a] Università degli Studi di Torino, Department of Agricultural, Forest and Food Sciences (DISAFA), Largo P. Braccini 2, 10095 Grugliasco (TO), Italy.

[b] Consiglio per la ricerca in agricoltura e l'analisi dell'economia agraria, Research Centre for Genomics and Bioinformatics, via San Protaso 302, 29017 Fiorenzuola d'Arda, Italy.

[c] Università degli Studi di Milano, Department of Food, Environmental and Nutritional Sciences (DeFENS), via G. Celoria 2, 20133 Milan, Italy.

[d] Consiglio per la ricerca in agricoltura e l'analisi dell'economia agraria, Research Centre for Cereal and Industrial Crops, S.S. 11 for Torino km 2,5, 13100 Vercelli, Italy.

*Corresponding author: Massimo Blandino

Phone +39 011 6708895, massimo.blandino@unito.it

SUPPLEMENTARY FIGURES

Figure 1S

Effect of FACE treatment on the relative occurrence of soluble (SPAs) and cell wall-bound (CWBPAs) phenolic acids in common wheat wholemeal.

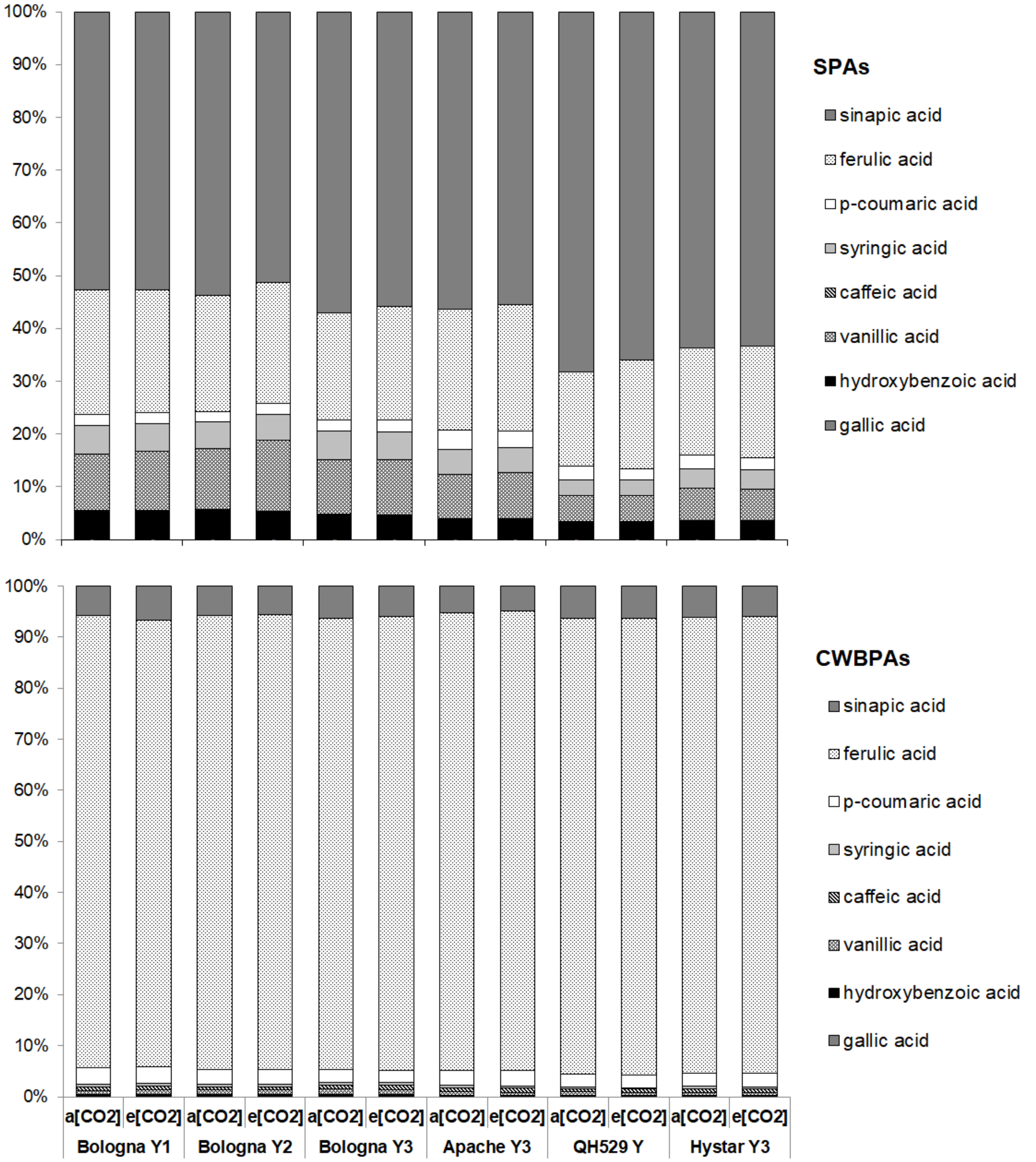

a[CO_2] = atmospheric carbon dioxide concentration, e[CO_2] = elevated carbon dioxide concentration

Experiment carried out in 3 years (Y1, 2011-12; Y2, 2012-13 and Y3, 2015-16).
